# Frontiers in Pediatric Urology – Specialty Grand Challenge

**DOI:** 10.3389/fped.2013.00015

**Published:** 2013-07-22

**Authors:** Ricardo González

**Affiliations:** ^1^Pediatric Surgery and Urology, Auf der Bult Kinder und Jugend KrankenhausHannover, Germany; ^2^Virchow Klinikum, Universitätsmedizin Berlin CharitéBerlin, Germany

“The greatest obstacle to discovery is not ignorance; it is the illusion of knowledge.”

Daniel J. Boorstin

“If I have seen a little further it is by standing on the shoulders of Giants.”

Isaac Newton

Pediatric urology is coming of age. Evidence of this is the success of specialty meetings, the increasing number of surgeons from around the world who practice predominantly or exclusively pediatric urology, and the fact that now there is more than one journal devoted exclusively to the field.

If we go back to the publication of what to my knowledge is the first textbook of pediatric urology by Meredith Campbell in 1937 (1), at a time when pediatric urology as an organized specialty did not exist (2), and fast forward to today when there is a plethora of books on the subject, regional pediatric urology societies, major associations in each continent with well attended congresses and dedicated journals, we can be proud to be part of this exciting process of maturation of this specialized body of knowledge.

Since my beginnings in urology, I have witnessed incredible technical advances in the operations to repair hypospadias, the popularization of intermittent catheterization which, revolutionized the management of neuropathic bladder dysfunction, progress in the surgical repair of major birth defects such as bladder exstrophy and persistent cloaca, application of endourological procedures to children, significant advancements in managing urinary incontinence, both medical and surgical and, in progress in postoperative pain control. Through cooperation with other disciplines, we have also seen significant advances in the treatment of genitourinary tumors and in pediatric renal transplantation. The development and application of minimally invasive surgery to children of all ages with genitourinary disorders represents one of the latest significant advancements (3).

Yet we have many other challenges ahead, among them, those discussed in the following sections need to be addressed by present and future generations of academic pediatric urologists.

## Clinical Challenges

Among the many clinical challenges faced by our specialty, I shall discuss only some, based more on my interests and experiences than on their objective significance.

### Role of fetal intervention

Prenatal diagnosis has revolutionized pediatrics and obstetrics. In places where prenatal ultrasound screening is done routinely, pediatric urologists are in a position to counsel the expecting parents on the possible diagnosis, treatment options, and prognosis for a given fetus. We can then act quickly after birth when indicated to prevent possible adverse effects of an unsuspected malformation. Despite the obvious advantages, prenatal diagnosis has had some adverse consequences as well. In the early days, it led to many unnecessary interventions, for example for fetal dilatation of the renal pelvis. Another consequence has been an increase in the number of terminations of pregnancy (4). While the decrease in the number of serious malformations may be a welcome benefit to society, many unjustified terminations have taken place and the resulting decrease in the number of certain malformations has been detrimental to the training of future generations of pediatric urologists. A fluid dialog between obstetricians and pediatric specialists such as I have experienced in some institutions where I worked needs to become widespread.

Initially there was great enthusiasm with the prospect of improving outcomes by intervening *in utero* or in the early post-natal period. Initial results although promising were below the expectations mostly due to technical limitations (5). Today, prenatal intervention for serious obstructive malformations such as posterior urethral valves or urethral atresia is done only exceptionally, although with conventional management the incidence of renal failure is high. It remains a challenge to re-explore this field in a systematic and scientific manner.

### Transitional urology

Not too many decades ago, the survival of children with serious malformations such as spina bifida with hydrocephalus or exstrophy of the cloaca was exceptional. In the last 50 years, infant mortality due to such malformations has become rare in the develop world. Thus, a significant number of individuals with long term need of medical and psychological care reach adulthood (6). Finding specialists capable and interested in treating these persons is problematic, particularly in environments in which circumstances do not allow pediatric specialists to care for grown up patients. A limited number of centers and individuals have taken this challenge but in general the urological care of adult patients who have had bladder exstrophy, spina bifida, or anorectal malformations to mention just a few remains problematic. This challenge is not limited to genitourinary diseases but also exists in other areas such as cardiology and neurosurgery. A system to train urologists able and willing to care for this patient population is needed in most parts of the world (7).

### Long term outcomes of anomalies with lifelong repercussions

Surgical techniques to treat numerous anomalies of the genitourinary system are constantly developed, modified, and perfected. A classic example is the correction of hypospadias, a genital malformation occurring with a frequency greater than 1:300 boys. More than 200 techniques are said to have been developed, many in the last four decades (8). New techniques are developed mainly to achieve better cosmetic results and decrease the number of re-intervention in the short term. Yet, publications of long term results (until the patient reaches adulthood) for new and old techniques are few and far between.

Surgeons tend to abandon old procedures and adopt new ones based on its apparent simplicity and presumed better results in what sometimes appears to be a trend to stay “modern and fashionable” rather than based on critical analysis of published results. For example, although past hypospadias repair accounts for a significant number of urethral strictures in adults (9) relatively few papers critically analyze urethral function after hypospadias repair. This is despite the fact that it is likely that living with a urethral stricture is more detrimental to quality of life than having small cosmetic imperfections in the appearance of the penis. I used hypospadias as an example of one of the most frequent anomalies treated by pediatric urologists but similar dilemmas exist in other types of genital reconstruction as well as in many other diseases.

### Prospective studies

Prospective studies, ideally with control groups and randomization are the backbone of evidence based medicine. Unfortunately, precious few such studies exist to back up management protocols in our specialty. The obstacles to performing such studies are great; nevertheless to move beyond where we are in the treatment of pediatric genitourinary diseases they are sorely needed. This is one of the greatest challenges for pediatric urologists. Given that in Western countries the numbers of cases is often too small to run such studies, two possible options to solve this problem come to mind. One is the conduction of multicenter studies and the other is setting up protocols for studies in countries with large populations treated at high volume centers. It is our job to set these options in motion. We should strive to make all treatment indications based on evidence. It is our responsibility to conduct research to provide such evidence.

## Research Challenges

Basic research conducted by scientists working in close collaboration with surgeons is the basic for significant progress in any surgical specialty. The ideal of the surgeon–scientist which was instilled in me at the onset of my training is probably unrealistic in most settings given the heavy clinical workloads dictated by third party payers. More realistic is to develop a close cooperation between surgeons providing the clinical perspective and full time laboratory researchers. The areas of bioengineering, genetics, mechanisms of disease are at the forefront of current research in pediatric urology. Academic surgeons should continue to lead the way for innovations and new understanding of diseases they treat.

In the US pediatric urology training programs there is often 1 year devoted to research. Whether or not this is an effective approach to stimulate young surgeons to pursue an academic and research career is open to question given time limitation and relative shortness of mentors (10). More effective is probably to provide interested faculty members adequate protected time to conduct research.

## Institutional Challenges

### Training in pediatric urology

Advances have been made in many countries formalizing the training requirements for future specialists. Different approaches have been taken in the US, Canada, and in Europe. In the US and Canada, opportunities for specialized training are limited to those who have completed training in general urology. A more encompassing approach in Europe allows both urologist and pediatric surgeons to pursue advanced training in pediatric urology increasing the number and spectrum of potential trainees. The decades ahead will reveal if one approach is superior to the other.

One of the challenges of training surgeons to treat rare anomalies is the relatively small number of complex cases at most institutions. This is particularly problematic in the Western world with large number of centers, low birth rates, and decreasing incidence of major malformations. Setting up training collaborations with large volume centers in countries with large populations and greater incidence of birth defects may be a solution to the problem. How to obtain funding and accredit such programs are obstacles to overcome.

Training and providing expert care for rare malformations are closely related. Another approach to provide training and expert care to children with rare congenital malformations is the creation of regional centers of expertise to which patients are referred, thus creating teams of expert surgeon–teachers in one particular malformation. This approach is being used in the United Kingdom for the treatment of bladder exstrophy, and could be expanded to other parts of the world and other diseases. The obstacles to this approach are transportation difficulties for patients and families in certain parts of the world, the financial interest of individual surgeons, and, last but not least are the surgeons’ egos. Keeping in mind the wellbeing of children, we should be able to overcome the first two obstacles in a relatively short time. This leads me to the discussion of the last topic of this article.

### Better integration of the groups

In some countries, pediatric surgeons and urologists taking care of children with genitourinary malformations and diseases work separate from each other, even in the same institution. Progress in our field could be accelerated by having better integration and cooperation between specialists with common interests regardless of their training background. The creation of Pediatric Urology units at major institutions would allow such integration. Geographical differences also determine who is better qualified to care for children with genitourinary disorders. In the US, rotations in pediatric urology are mandatory in the course of training in urology so that a few individuals become interested in pursuing further training. In contrast in Europe, many trainees in urology have minimal or no exposure to pediatric urology whereas many pediatric surgery training programs include rotations in urology (11).

Also greater cooperation between developed and developing countries would be beneficial. Although the efforts of groups and individuals traveling from the developed world to needy regions to operate on children is beneficial to individuals, greater benefit could be gained by using experienced surgeons to train local teams to care for their children on more permanent basis. Isolated examples of this model exist in places like Eritrea (12) and Pakistan (Sultan S, personal communication).

Another related issue that needs emphasis is that pediatric urology cannot exist in a vacuum. Good cooperation with pediatric nephrologists, endocrinologists, pediatric imaging specialists, psychologists, neonatologists, and other pediatric specialties is essential to provide state of the art care.

## Social Challenges

The role of the pediatric urologist in the pediatric health care system has evolved differently in various environments. For example, earlier on in the USA, most pediatric urologists were attached to academic medical centers and involved in complex surgical reconstructive procedures. As the numbers of practitioners increased and more moved to private or non-academic settings, they become involved in more routine procedures previously in the hands of general urologists or general pediatric surgeons. Simultaneously their involvement in the management of non-surgical conditions such as non-neuropathic voiding disorders, otherwise in the domain of the general pediatrician increased. In many settings, the role of physician's extenders has become crucial to the fulfilling of all the clinical demands facing the pediatric urologist (13). The question of future manpower needs remains unresolved (10).

Outside the USA where there tends to be fewer specialists, their role continues to be predominantly as highly specialized surgeons in tertiary referral centers. As number of major cases per surgeon in the US decreases and practice shifts more toward the minor and routine cases (14), we should be concerned about the consequences of this trend upon surgical skills and training repercussions. Countries where pediatric urology is in earlier stages of development should learn from the US experience as they shape the future role of specialists. It would seem wiser for pediatric urologists to allow less specialized physicians continue care of the more common problems such as phimosis, undescended testis, and hernias which require less demanding skills and to involve non-surgeons for the care of voiding functional disorders.

## Conclusion

Pediatric Urology is coming of age and it is our challenge to make sure the transition to maturity is as successful as its development until now. We also must strive to distinguish what we know from what we think we know and to take advantage of our elevated vantage point to trace the future course of the specialty.
